# Risk factors associated with lymph node metastasis in cN0 papillary thyroid microcarcinoma

**DOI:** 10.3389/fonc.2026.1839787

**Published:** 2026-08-11

**Authors:** Shijie Zheng, Chuangwu Ke, Weijing Hao, Xiaohan Chen, Wenchao Zhang

**Affiliations:** 1 Department of Head and Neck Oncology, Tianjin Cancer Hospital Airport Hospital, National Clinical Research Center for Cancer, Tianjin, China; 2 Department of Head and Neck Oncology, Tianjin Medical University Cancer Institute & Hospital, Key Laboratory of Basic and Translational Medicine on Head & Neck Cancer, National Clinical Research Center for Cancer, Tianjin Key Laboratory of Cancer Prevention and Therapy, Tianjin’s Clinical Research Center for Cancer, Tianjin, China

**Keywords:** active surveillance, BRAF V600E, lymph node metastasis, neutrophil-to-lymphocyte ratio, papillary thyroid microcarcinoma

## Abstract

**Introduction:**

Although active surveillance (AS) is increasingly recommended for low risk papillary thyroid microcarcinoma (PTMC), a subset of patients with clinically node-negative (cN0) disease actually harbor occult lymph node metastasis (LNM), which may lead to delayed surgical intervention and poorer outcomes. This study aimed to identify independent risk factors for LNM in cN0 PTMC patients to refine preoperative risk stratification and guide individualized treatment decisions.

**Methods:**

We retrospectively analyzed 2097 cN0 PTMC patients who underwent thyroidectomy with central lymph node dissection at Tianjin Cancer Hospital Airport Hospital between September 2019 and March 2025. Preoperative clinical, ultrasonographic, and laboratory data were collected. Univariate and multivariate logistic regression analyses were performed to identify independent predictors of LNM. Receiver operating characteristic (ROC) curve analysis was used to evaluate predictive efficacy and determine optimal cut-off values.

**Results:**

Postoperative LNM was confirmed in 865 patients (41.2%). Multivariate analysis identified male sex (OR = 2.108, 95% CI: 1.632-2.723), younger age (OR = 0.964, 95% CI: 0.954-0.974), larger tumor diameter (OR = 4.001, 95% CI: 2.329-6.871), multifocality (OR = 1.300, 95% CI: 1.037-1.629), BRAF V600E mutation (OR = 8.938, 95% CI: 6.900-11.579), and higher neutrophil-to-lymphocyte ratio (NLR) (OR = 11.158, 95% CI: 8.221-15.138) as independent risk factors for LNM (all *p* < 0.05). ROC analysis revealed optimal cut-off values of ≤43.5 years for age, ≥0.65 cm for tumor diameter, and ≥1.925 for NLR. BRAF V600E mutation and NLR demonstrated the highest predictive efficacy (AUC = 0.720 and 0.759, respectively).

**Conclusions:**

In cN0 PTMC patients, male sex, age ≤43.5 years, tumor diameter ≥0.65 cm, multifocality, BRAF V600E mutation, and NLR ≥1.925 are independently associated with increased risk of cervical LNM. Preoperative assessment of these factors enables refined risk stratification for patients being considered for AS, facilitating more precise and individualized surveillance strategies. These findings underscore the importance of integrating clinical, pathological, and molecular parameters in PTMC risk stratification. Future prospective studies are warranted to validate whether early surgical intervention confers superior survival benefits over continued AS in high risk subgroups.

## 1 Introduction

Thyroid cancer (TC) stands as the most prevalent malignant tumor within the head, neck, and endocrine systems. Despite the fact that its overall mortality rate remains lower compared to other cancer types, the global incidence of TC has been steadily escalating, attributable to advancements in diagnostic technologies. Currently, TC accounts for 2.1% of all cancer cases, and notably, its incidence is significantly higher among females than males (1). The majority of thyroid cancers fall under the category of differentiated thyroid cancer (DTC). Among these, papillary thyroid carcinoma (PTC) is the most common, constituting approximately 85% of all cases. PTC typically demonstrates an indolent biological behavior and is associated with a favorable prognosis (2). However, it often presents with early regional lymph node metastasis, occurring in 30% - 80% of cases (3). Lymph node metastasis is closely associated with an elevated risk of adverse prognosis. Regarding patients with papillary thyroid microcarcinoma (PTMC) where the tumor diameter is ≤1 cm, since the majority exhibit no obvious clinical symptoms in the early stages, to prevent overtreatment, some scholars have proposed that active surveillance (AS) can serve as a rational alternative strategy to surgical treatment (4, 5), the guidelines of the American Thyroid Association (ATA) in 2025 have explicitly indicated that for patients with low risk PTMC, AS represents a viable management strategy (6). Nevertheless, not all patients are amenable to the AS approach. study has indicated that within the PTMC patient population, 12.0% - 59.2% still experience lymph node metastasis (7–9). In some cases, preoperative imaging examinations fail to detect metastatic lesions, leading to misclassification as low risk. This misjudgment consequently delays the optimal surgical intervention, potentially exerting a detrimental impact on long-term survival. Therefore, although PTMC generally poses a relatively low risk, its heterogeneity demands due consideration. The ability to accurately predict, prior to surgery, those PTMC patients with actual lymph node metastasis despite negative imaging findings (cN0) would significantly facilitate the formulation of more precise and individualized treatment regimens.

## 2 Materials and methods

### 2.1 Patient selection

Clinical data of all patients hospitalized for the treatment of thyroid cancer between September 2019 and March 2025 were retrospectively retrieved from the electronic medical record system of our hospital.

### 2.2 Inclusion criteria

1. Patients who underwent unilateral or total thyroidectomy and concurrent central lymph node dissection;
2. Preoperative fine needle aspiration (FNA) cytology or postoperative pathology confirmed PTC, with the maximum tumor diameter ≤ 1 cm;
3. No lymph node metastasis was detected by preoperative color Doppler ultrasound, CT or other imaging examinations;
4. Complete baseline clinical data were available.

### 2.3 Exclusion criteria

1. Patients who did not undergo lymph node dissection;
2. Patients whose pathological types are concurrent with other types of thyroid cancers (e.g., follicular carcinoma, medullary carcinoma, undifferentiated carcinoma, or poorly differentiated carcinoma) or other primary malignancies;
3. Patients with distant metastases;
4. Patients who had received radiotherapy or chemotherapy prior to surgery;
5. Patients who were not undergoing their first thyroid cancer surgery or had a recurrence of thyroid cancer;
6. Patients with hematological disorders, acute infections within two weeks prior to surgery, or those using corticosteroids, immunosuppressants, or other agents that may interfere with white blood cell and lymphocyte counts.
7. Patients with mental disorders or cognitive impairments;
8. Patients with severe organ dysfunction (involving vital organs such as the brain, heart, lungs, liver, and kidneys);
9. Pediatric patients.

### 2.4 Data collection

This study undertook a retrospective analysis of the preoperative and postoperative clinical data of patients. The vast majority of patients were diagnosed via ultrasound examination, which was carried out by well experienced ultrasound diagnosticians. Subsequently, the results were reviewed by another chief ultrasound physician, and re-evaluated by surgeons prior to surgery. The main aspects of assessment encompassed the location, quantity, maximum diameter, calcification status, morphological characteristics, and blood flow distribution of thyroid nodules. Relevant imaging data were systematically documented. Extrathyroidal Extension (ETE) was defined as the situation where the tumor penetrated the thyroid capsule and invaded surrounding soft tissues, including muscles, trachea, nerves, or blood vessels. The determination of ETE was based on the findings of postoperative pathological analyses. The diagnosis of Hashimoto’s Thyroiditis (HT) relied on immunological assays or postoperative pathological findings. Specifically, if the titers of thyroid globulin antibody and thyroid peroxidase antibody in the patient’s serum were both significantly elevated, exceeding 50% of the upper limit of the normal reference range, HT was diagnosed. The mutation status of the BRAF V600E gene was ascertained through either preoperative fine needle aspiration biopsy or postoperative pathological histological tests. The levels of thyroid stimulating hormone (TSH), thyroid globulin (Tg), and the neutrophil-to-lymphocyte ratio (NLR) (10), were obtained from preoperative laboratory tests.

### 2.5 Statistical methods

Statistical analysis was performed using SPSS 26.0 software. The normality of measurement data was determined by the Kolmogorov-Smirnov test: measurement data that conformed to a normal distribution were expressed as mean ± standard deviation (x ± s), and the comparison between groups was conducted using the independent samples T-test; measurement data that did not conform to a normal distribution (skewed) were expressed as median and interquartile range [M (P_25_ - P_75_)], and the comparison between groups was conducted using the Mann-Whitney U test. Count data were expressed as frequency and percentage [n (%)], and the comparison between groups was conducted using Chi-square test. The relevant factors with *p* < 0.05 in the univariate analysis were incorporated into the binary logistic regression analysis to identify the independent risk factors influencing the prognosis of patients. The predictive efficacy of each clinical indicator for lymph node metastasis in papillary thyroid microcarcinoma without preoperative indication of lymph node metastasis was evaluated by drawing the receiver operating characteristic (ROC) curve and calculating the area under the curve (AUC). *p* < 0.05 was considered statistically significant.

This study was approved by the Ethics Committee of Tianjin Cancer Hospital Airport Hospital.

## 3 Result

**Figure 1:**
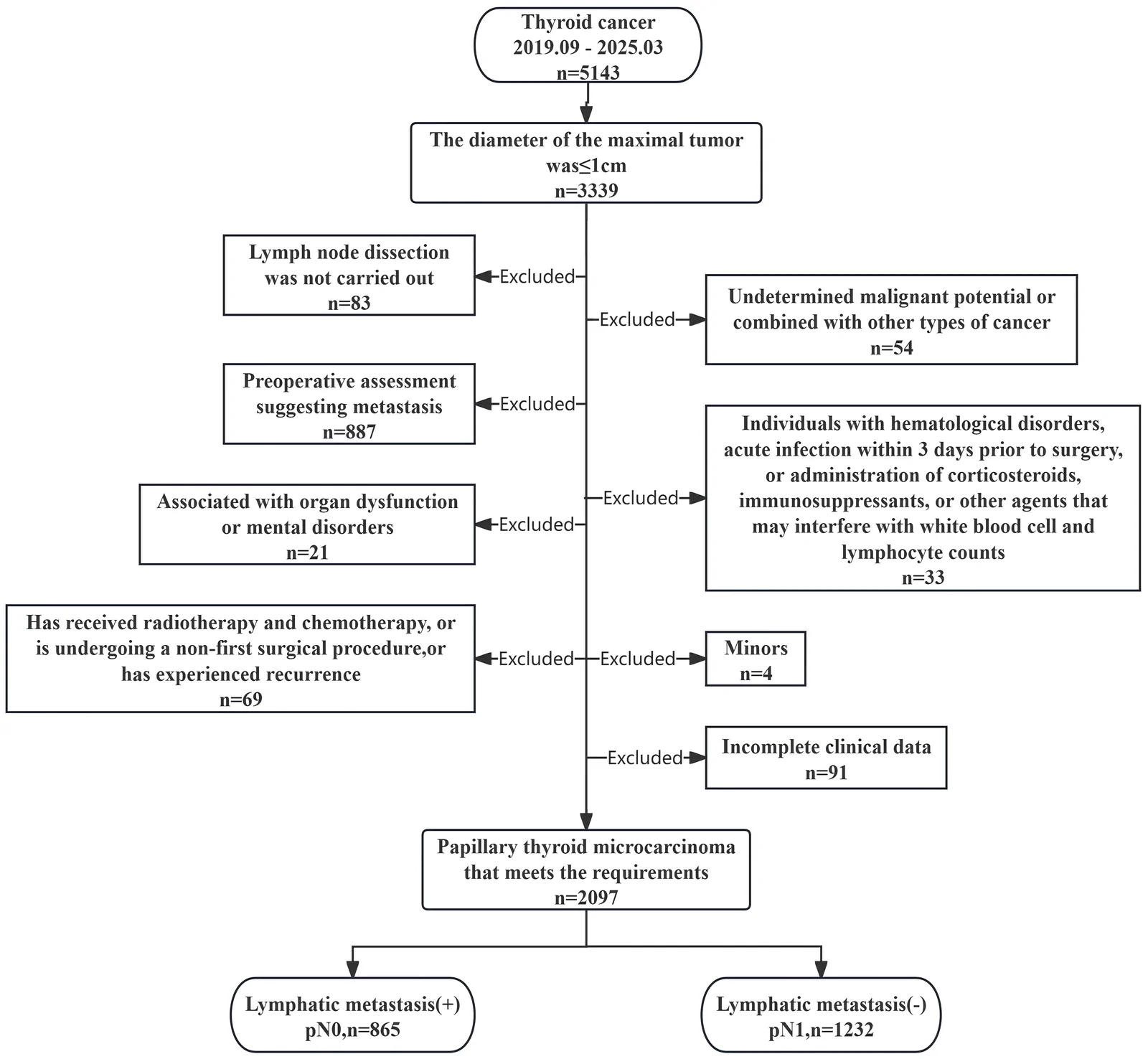
Flow diagram of the research subjects.

This study retrospectively enrolled 5143 patients with PTC who had received surgical treatment at our hospital between September 2019 and March 2025. Among them, 3339 cases had a maximum tumor diameter of ≤ 1 cm. After being screened according to strict exclusion criteria, 2097 patients with cN0 PTMC were ultimately included in the study. For the detailed screening process, refer to **Figure 1**.

### 3.1 Analysis of clinical baseline characteristics in patients with cN0 PTMC

A total of 2097 patients were included in this study. Among them, 865 cases (41.2%) had lymph node metastasis, and 1232 cases (58.8%) had no lymph node metastasis. There were 514 male patients (24.5%) and 1583 female patients (75.5%). The median age was 44 years (interquartile range: 36–53), and the median maximum tumor diameter was 0.7 cm (interquartile range: 0.5–0.8). The tumors were located in the upper part of the thyroid gland in 743 cases (35.4%), in the middle part in 736 cases (35.1%), and in the lower part in 618 cases (29.5%). There were 1287 cases (61.4%) with single lesions and 810 cases (38.6%) with multiple lesions. Lesions were confined to one side in 1545 cases (73.7%) and involved both sides in 552 cases (26.3%). Calcification was observed in 1816 cases (86.6%) by imaging or pathological examination, and no calcification was found in 281 cases (13.4%). There were 144 cases (6.9%) with ETE, and 1953 cases (93.1%) without ETE. There were 529 cases (25.2%) with HT and 1568 cases (74.8%) without it. The median TSH level was 2.03 uIU/mL (interquartile range: 1.25–3.45), and the median Tg level was 60.5 μg/L (interquartile range: 37.29–83.68). There were 1293 cases (61.7%) carrying BRAF V600E mutation and 804 cases (38.3%) without it. The median NLR was 2.11 (interquartile range: 1.79–2.43).

### 3.2 Comparison of clinical baseline data between the lymph node metastasis group and the non - metastasis group

In this study, a total of 2097 patients with cN0 PTMC were enrolled. Based on the postoperative pathological findings, they were classified into a lymph node metastasis group (pN1) consisting of 865 patients (accounting for 41.2%) and a non - lymph node metastasis group (pN0) with 1,232 patients (58.8%).

Appropriate statistical methods were employed for different types of variables. The results of univariate analysis indicated that, in comparison to the N0 group, the N1 group exhibited a higher male proportion (32.4% vs. 19.0%, *p* < 0.001), a younger age (41 years old vs. 47 years old, *p* < 0.001), a larger tumor diameter (0.7 cm vs. 0.6 cm, *p* < 0.001), a greater prevalence of multifocal lesions (41.8% vs. 36.4%, *p* = 0.011), a higher BRAF V600E mutation rate (87.5% vs. 43.5%, *p* < 0.001), and a significantly elevated NLR (2.36 vs. 1.90, *p* < 0.001).

Conversely, regarding aspects such as tumor location, bilaterality, calcification status, the presence or absence of HT, TSH level, Tg level, and ETE, no statistically significant differences were observed between the two groups (*p* > 0.05). Specific details are presented in **Table 1**.

**Table 1:** Univariate analysis demonstrated the association between the clinical data of patients with cN0 PTMC and lymph node metastasis.

Factor		Lymphatic metastasis(+) pN1, n=865(41.2%)	Lymphatic metastasis(-) pN0, n=1232(58.8%)	*P* value
Gender	Male	280(13.4%)	234(11.2%)	<0.001
	Female	585(27.9%)	998(47.6%)	
Age(years)		41(34-50)	47(38-54)	<0.001
Tumor diameter(cm)		0.7(0.6-0.9)	0.6(0.5-0.8)	<0.001
Location	Upper	311(14.8%)	458(21.8%)	0.480
	Top	297(14.2%)	392(18.7%)	
	Lower	257(12.3%)	382(18.2%)	
Multifocality	Yes	362(17.3%)	448(21.4%)	0.011
	No	503(24.0%)	784(37.4%)	
Bilateral	Yes	242(11.5%)	310(14.8%)	0.150
	No	623(29.7%)	922(44.0%)	
Calcification	Yes	763(36.4%)	1053(50.2%)	0.070
	No	102(4.9%)	179(8.5%)	
ETE	Yes	57(2.7%)	87(4.2%)	0.674
	No	808(38.5%)	1165(54.6%)	
HT	Yes	209(10.0%)	320(15.2%)	0.347
	No	656(31.3%)	912(43.5%)	
TSH(uIU/mL)		1.98(1.26-3.45)	2.04(1.24-3.44)	0.791
Tg(μg/L)		60.24(37.16-82.61)	61.01(37.27-84.29)	0.439
BRAF V600E	Yes	757(36.1%)	536(25.6%)	<0.001
	No	108(5.1%)	696(33.2%)	
NLR		2.36(2.07-2.59)	1.90(1.65-2.23)	<0.001

### 3.3 Binary logistic regression analysis was performed to explore in greater depth the independent risk factors for lymph node metastasis

The findings of univariate analysis revealed a significant association between gender, age, tumor diameter, multifocality, the mutation status of BRAF V600E, and NLR and lymph node metastasis (*p* < 0.05).

Upon incorporating these variables into the binary logistic regression model, the analysis demonstrated that gender (odds ratio [OR] =2.108, 95% confidence interval [CI]: 1.632-2.723, *p* < 0.001), age (OR = 0.964, 95% CI: 0.954-0.974, *p* < 0.001), tumor diameter (OR = 4.001, 95% CI: 2.329-6.871, *p* < 0.001), multifocality (OR = 1.300, 95% CI: 1.037-1.629, *p* = 0.023), BRAF V600E mutation (OR = 8.938, 95% CI: 6.900-11.579, *p* < 0.001), and NLR (OR = 11.158, 95% CI: 8.221-15.138, *p* < 0.001) were all independent determinants of lymph node metastasis. Specific details are presented in **Table 2**.

**Table 2:** Binary logistic regression analysis was employed to further validate the independent risk factors for lymph node metastasis.

Relevant factor	Odds ratio(OR) [95% confidence interval (CI)]	*P* value
Gender	2.108 (1.632 - 2.723)	<0.001
Age	0.964 (0.954 - 0.974)	<0.001
Tumor diameter	4.001 (2.329 - 6.871)	<0.001
Multifocality	1.300 (1.037 - 1.629)	0.023
BRAF V600E	8.938 (6.900 - 11.579)	<0.001
NLR	11.158 (8.221 - 15.138)	<0.001

### 3.4 The predictive efficacy of independent risk factors was evaluated via ROC curve analysis, and the optimal cut-off value was determined

In order to further assess the predictive significance of gender, age, tumor diameter, multifocality, BRAF V600E mutation status, and NLR regarding lymph node metastasis in patients with PTMC, this study employed ROC curve analysis.

**Figure 2:**
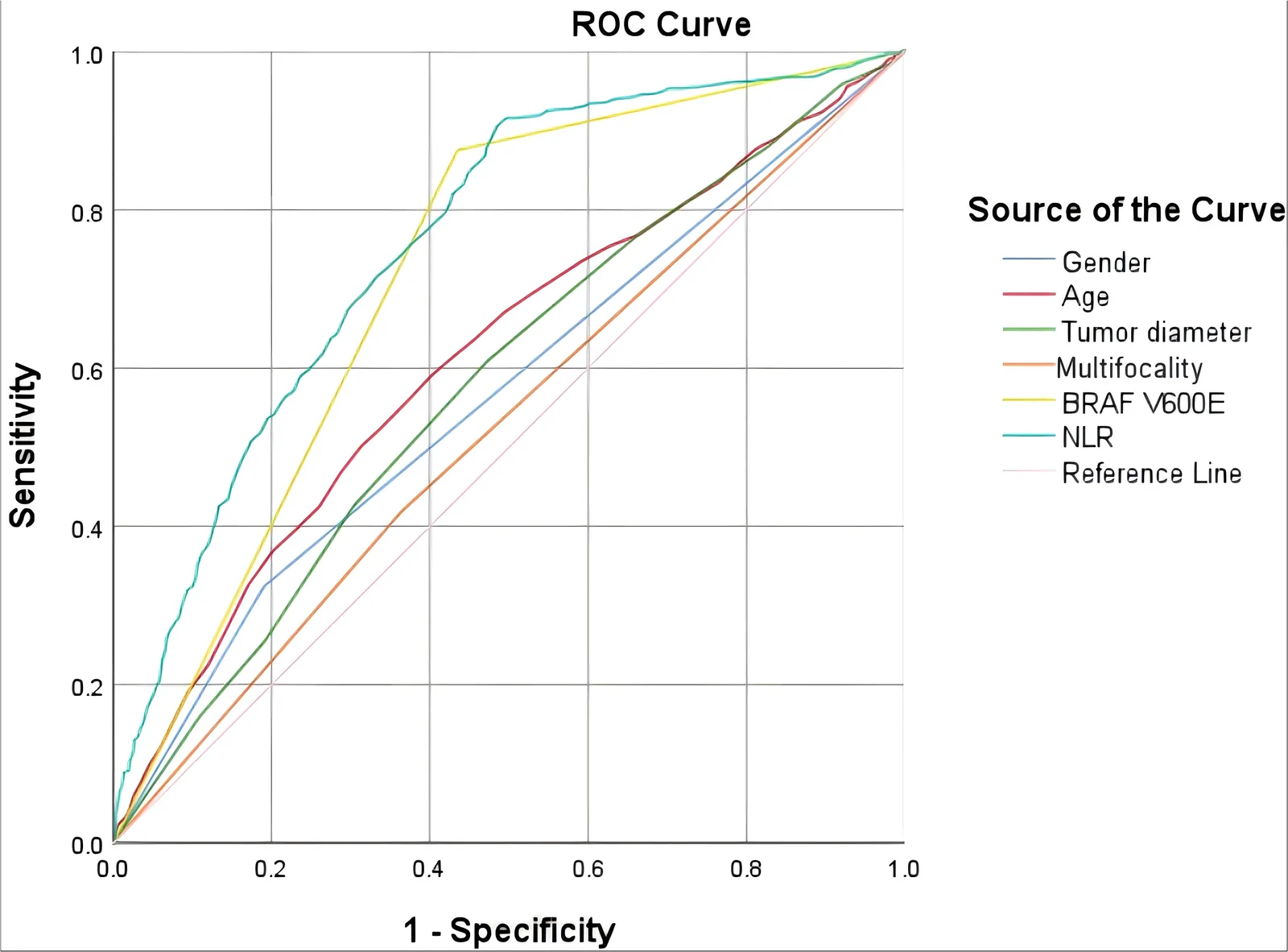
Predictive efficacy of gender, age, tumor diameter, multifocality, BRAF V600E and NLR for lymph node metastasis.

The area under the curve (AUC) for gender was 0.567 (95% CI: 0.542-0.592, *p* < 0.001), indicating that male patients were more prone to lymph node metastasis. For age, the AUC was 0.614 (95% CI: 0.589-0.639, *p* < 0.001). The optimal cut-off value was ascertained by calculating the Youden index (Youden index = sensitivity + specificity - 1). The maximum value of the Youden index was 0.189, corresponding to an optimal age cut off of 43.5 years, with a sensitivity of 0.590 and a specificity of 0.599. This implies that patients with an age of ≤ 43.5 years had a higher likelihood of lymph node metastasis. The AUC for the tumor diameter was 0.581 (95% CI: 0.556-0.606, *p* < 0.001). The maximum value of the Youden index was 0.136, corresponding to an optimal cut-off value of 0.65 cm, with a sensitivity of 0.609 and a specificity of 0.527. This suggests that when the tumor diameter was ≥ 0.65 cm, the risk of lymph node metastasis increased. The AUC for multifocality was 0.527 (95% CI: 0.502-0.553, *p* = 0.032), suggesting that patients with multifocal lesions were more likely to experience lymph node metastasis. Regarding the BRAF V600E mutation, the AUC was 0.720 (95% CI: 0.698-0.742, *p* < 0.001), indicating that patients carrying this mutation had a substantially elevated risk of lymph node metastasis. For NLR, the AUC was 0.759 (95% CI: 0.739-0.780, *p* < 0.001). The maximum value of the Youden index was 0.42, corresponding to an optimal cut-off value of 1.925, with a sensitivity of 0.905 and a specificity of 0.515. This indicates that when NLR was ≥ 1.925, the probability of lymph node metastasis was higher. As depicted in **Figure 2**.

## 4 Discussion

In the realm of thyroid cancer treatment strategies, no unified consensus has been established across different disciplines over an extended period. The question of whether patients should receive surgical intervention remains a central point of clinical contention. With the emergence of the concept of “overdiagnosis and overtreatment,” an increasing number of countries have adopted AS as a management approach for patients with low risk PTMC (4, 5). However, not all PTMC cases follow an indolent course. A subset of patients may exhibit clinical progression within a short timeframe, including an increase in tumor volume, lymph node metastasis, or ETE (11). Delaying surgery until the disease reaches an advanced stage may heighten the risk of poor prognosis, resulting in elevated recurrence rates and a higher incidence of distant metastases (12). Additionally, ultrasonography has a relatively limited sensitivity, ranging from 28% to 39% (13), in detecting lymph node metastases through imaging. Relying solely on ultrasonic imaging for risk stratification is evidently insufficient. Despite this, the potential negative impact of excessive surgical interventions on patients’ quality of life should not be overlooked. Thus, accurately identifying cN0 PTMC patients who genuinely require surgical intervention has emerged as a crucial clinical issue demanding immediate attention.

Numerous previous studies have indicated that gender exerts a crucial influence on disease progression, which is in line with the findings of our current study. Although the incidence of PTMC is higher in females, male patients are more prone to tumor progression and have been demonstrated to be an independent risk factor for lymph node metastasis in PTMC (14). This disparity might be associated with the relatively high androgen levels or low estrogen levels in males. Such an endocrine milieu may facilitate the invasive behavior of tumors and lymph node metastasis.

Despite the differences in the definition of age cut off points across various studies, numerous studies have established age as an independent risk factor for the progression of PTMC (15, 16). A retrospective analysis revealed that the disease progression rate was 8.9% among patients under 40 years of age, 3.5% in the 40–59 year old group, and 1.6% in individuals aged 60 years or older (17). This finding suggests that younger patients are more likely to experience clinical progression. In our study, it was demonstrated that patients aged ≤ 43.5 years exhibit a significantly higher risk of lymph node metastasis. This observation is in line with the trends reported in the aforementioned studies.

Tumor size serves as a crucial metric for assessing the progression of PTMC. Extensive research has firmly established a significant correlation between tumor diameter and the risk of lymph node metastasis, indicating that larger tumors are more predisposed to metastasis (18). This finding aligns seamlessly with the outcomes of our present study. In our study, we calculated the Youden index to determine the optimal cutoff value. The results unambiguously demonstrate that when the tumor diameter reaches ≥ 0.65 cm, the risk of lymph node metastasis escalates substantially.

The role of multifocality in PTC remains a debated topic. Multifocal tumors can arise either from the spread of a single primary tumor within the gland or from multiple synchronous primary tumors, with the latter being the most common cause (19). Most studies have suggested that multifocality does not impact the progression of thyroid cancer. However, some scholars have indicated that multifocality is a factor associated with lymph node metastasis in PTC (20). In our study, we discovered that multifocality is an independent influencing factor for cN0 PTMC. Moreover, some research has shown that the risk of recurrence increases as the number of cancer foci rises. Furthermore, some scholars have found that there is no significant difference in the recurrence risk between bilateral multifocality and unilateral multifocality. This suggests that multifocality, rather than bilateral, may be the cause of poor prognosis in PTC (21), which is consistent with the findings of our study.

The BRAF V600E mutation, which results from the substitution of valine (V) at position 600 with glutamic acid (E), is a relatively common molecular alteration in PTC. In this study, the BRAF V600E mutation was confirmed as an independent risk factor for lymph node metastasis in cN0 PTMC, a finding consistent with the conclusions of many previous studies. Previous research has shown that the BRAF V600E mutation is significantly associated with larger tumor diameter (≥0.5 cm but <1 cm), multifocality, ETE, lymph node metastasis, advanced disease stage, and increased risk of recurrence (22). These findings suggest that PTC carrying the BRAF V600E mutation may not exhibit the “indolent tumor” behavior traditionally believed, but should be regarded as a more aggressive subtype, warranting more aggressive clinical intervention strategies. Additionally, during the data collection process, we observed that for some patients who underwent FNA, although cytology did not suggest malignancy, if the BRAF V600E mutation was detected, the postoperative paraffin pathological examination results almost confirmed malignancy (only 1 case did not return a malignant result), this finding further highlights the potential value of BRAF V600E detection in preoperative risk stratification and diagnosis.

The progression of tumors can be mediated by inflammatory cells. Among these, neutrophils play a crucial role in the initiation, development, and angiogenesis of tumors. They achieve this by inhibiting the secretion of tumor necrosis factor and promoting the production of vascular endothelial growth factor, thus facilitating tumor invasion. On the other hand, tumors may also disrupt the balance of the immune system. Lymphocytes, as a vital component of the immune system, can induce the activation of natural killer cells and stimulate macrophages to release interferon and tumor necrosis factor, thereby exerting an anti - tumor effect. This helps to inhibit tumor growth and reduce tumor volume (23). The NLR, as a preoperative hematological biomarker, offers the advantages of objectivity, accuracy, and easy accessibility. Its prognostic value in various malignant tumors has been confirmed by numerous studies (24). Numerous investigations have demonstrated that there exists a substantial disparity in the levels of NLR between patients with PTC at the early stage (I/II) and those at the advanced stage (III/IV). Specifically, NLR has been found to be correlated with the 5 year disease free survival rate of patients. This association implies that NLR could potentially serve as a prognostic biomarker for assessing the disease risk of PTC (25). The findings of this study indicate that NLR is significantly correlated with lymph node metastasis in cN0 PTMC. The ROC curve analysis reveals that the AUC of NLR is 0.759, which is higher than that of other clinical indicators. Further calculations based on the Youden index suggest that when NLR ≥ 1.925, the risk of lymph node metastasis in patients increases significantly.

Furthermore, no significant association between tumor location and lymph node metastasis was observed in the present study. In contrast, certain pathological features (e.g., capsular invasion, vascular tumor thrombus) and molecular markers (e.g., BRAF V600E mutation) may serve as more robust predictors of lymph node metastasis. Within this study cohort, the aforementioned high-risk factors were prevalent, and their strong predictive effects likely masked the potential impact of tumor anatomical location on metastatic risk. While HT has been identified as a protective factor in several prior studies (14, 26), no such association was detected herein. This discrepancy may stem from differences in diagnostic criteria: some investigations relied on preoperative serological indices (e.g., elevated TPOAb/TgAb levels) for diagnosing HT, whereas the present study employed pathological diagnosis as the gold standard. Additionally, the frequency of BRAF V600E mutations was relatively high in this cohort; this driver mutation may dominate the invasive and metastatic behavior of tumors, resulting in persistently high lymph node metastasis rates regardless of concurrent HT. Notably, ETE was also not significantly correlated with lymph node metastasis in the present study, a finding that contradicts prevailing clinical perceptions. A plausible explanation is that the study population may encompass two tumor subtypes with distinct biological behaviors: one consisting of early-stage lesions with high lymphatic metastatic potential that have not yet breached the glandular capsule, and another comprising indolent tumors with mild ETE but low metastatic activity. Such heterogeneity may have diluted the overall statistical association between ETE and lymph node metastasis. Future studies incorporating subtype-specific analyses may uncover more precise clinicopathological correlations. Mechanistically, lymph node metastasis is a multi-step, complex biological process. Some high-grade tumors may preferentially disseminate via hematogenous routes, or their metastatic behavior may be primarily driven by specific molecular alterations (e.g., BRAF V600E mutation, TERT promoter mutation). These intrinsic mechanisms may override the predictive value of ETE as a single anatomical parameter. Thus, the data from this study suggest that ETE may not be a reliable independent indicator for assessing lymph node metastasis risk in specific patient subgroups, particularly those with localized microextrathyroidal invasion but indolent biological behavior. This finding underscores the importance of integrating multiple clinical, pathological, and molecular parameters in thyroid cancer risk stratification and reflects the high heterogeneity of invasion patterns in thyroid cancer, warranting further validation in larger-scale prospective studies.

Despite the aforementioned negative findings, the clinical imperative to accurately identify occult lymph node metastases remains pressing. The challenge of precisely predicting occult lymph node involvement in cN0 patients is not confined to PTMC but also represents a recurring dilemma in head and neck squamous cell carcinoma(HNSCC). This parallel endows our research findings with enhanced translational relevance. In a large scale multicenter “real world” cohort study encompassing 2307 patients with laryngeal and hypopharyngeal squamous cell carcinoma, Knopf et al. demonstrated that for early stage (T1/2N0M0) tumors, surgical treatment yielded overall survival outcomes comparable to those of conservative management. However, in lymph node positive cases, surgery significantly improved survival rates (27). This observation underscores the pivotal role of accurate lymph node staging in determining patient prognosis, suggesting that misclassification of lymph node positive patients as cN0 may inadvertently deprive them of the survival benefits conferred by timely surgical intervention. Other studies have further validated this concern: exclusive reliance on preoperative imaging modalities (CT/MRI and ultrasound) for lymph node assessment is inherently limited, potentially resulting in either overstaging or understaging (28). Our study extends this critical concept to PTMC management—both conditions share fundamental parallels regarding the uncertainty of lymph node staging and the potential impact of nodal status on treatment decision-making. Grounded in this shared understanding, we endeavor to construct a refined risk stratification model for cN0 PTMC patients by integrating clinical, pathological, and molecular parameters. This model aims to address the limitations of imaging-only assessment, providing objective evidence for patient selection and adjustment of follow-up intensity under AS strategies. For patients with multiple high risk factors, consideration may be given to shortening the interval between ultrasound follow-ups, or to early reassessment of AS feasibility and evaluation of the necessity for early surgical intervention. Conversely, low risk patients can be more confidently included in AS management. This risk-adapted stratified management strategy aligns with the clinical rationale in HNSCC, where selective neck dissection is guided by the risk of lymph node metastasis. It holds promise for advancing PTMC active surveillance from a “one size fits all” approach toward a more individualized and precision-oriented paradigm.

Several limitations should be acknowledged in the present study. First, as a retrospective observational study, it is susceptible to selection bias; moreover, the study cohort was recruited exclusively from a single medical institution, and thus the external validity of the findings to populations in other regions warrants further validation. Second, the current study lacks long-term follow-up data regarding the prognosis of patients with lymph node metastasis, leaving unresolved the question of whether there exists a significant difference in long-term survival rates between cN0 patients with lymph node metastasis and those without. Additionally, disparities in surgical techniques, variations in equipment, environmental exposures, and cultural contexts across different populations may compromise the generalizability of the study results. To address the aforementioned limitations and translate research findings into clinical practice, there is an urgent need for future prospective, multicenter studies with extended follow-up. These studies should aim to validate our risk stratification model across diverse populations, clarify whether early surgical intervention is superior to AS in high risk patients, and optimize the inclusion criteria for AS as well as the optimal timing of conversion to surgery. Such efforts are of critical importance for advancing the precision management of cN0 PTMC.

## 5 Conclusions

This study demonstrates that in patients with cN0 PTMC, male sex, age ≤43.5 years, maximum tumor diameter ≥0.65 cm, multifocality, BRAF V600E mutation positivity, and NLR ≥1.925 are independently associated with an elevated risk of cervical lymph node metastasis. These readily accessible preoperative parameters can serve as a refined basis for risk stratification in patients considering AS. For patients with multiple high risk factors, shortening the interval of ultrasound follow-up or conducting an earlier reassessment of the feasibility of AS may be considered, thereby providing a reference for formulating individualized monitoring protocols. Future prospective studies are required to further validate whether early surgical intervention can yield superior survival benefits over continued AS in this high risk subgroup.

## Data Availability

The raw data supporting the conclusions of this article will be made available by the authors, without undue reservation.
